# Three thousand years in Tibet

**DOI:** 10.1093/nsr/nwz172

**Published:** 2019-11-13

**Authors:** Bettina Harr, Trevor Price

**Affiliations:** 1 Max-Planck-Institut für Evolutionsbiologie, Germany; 2 Department of Ecology and Evolution, University of Chicago, USA; 3 Reviewer of NSR

Humans have come to inhabit nearly every terrestrial environment. Their commensals have followed them and have consequently become some of the most abundant and widespread species on Earth, in the process becoming exposed to a variety of abiotic and biotic stressors. For example, the house sparrow was introduced from the UK to North America in the nineteenth century and now millions of birds breed in houses from northern Canada to Tierra del Fuego. Clines in both morphology and colour were rapidly established [1]. But what processes cause these clines? One point of special interest is the extent to which this rapidly established geographical variation is a result of genetic evolution or plastic responses, because this has major implications for how species will respond to—for example—climate change [2].

In the UK, house sparrows live in houses and tree sparrows live in trees but, in east Asia, the house sparrow is absent and tree sparrows live in houses. Around 3600 years ago, humans established permanent residence on the high Tibetan plateau. Using genomics, Qu *et al.* [3] found that the date of arrival of the tree sparrow at these altitudes roughly coincides with that time, that sparrow effective population sizes are large in both the Chinese lowlands and Tibet, and that there is very little genome-wide differentiation between these locations. They also show that flight muscles are larger in Tibet than in the lowlands, which may be a response to cold and/or to hypoxia. The large population sizes and lack of general differentiation mean that drift is not expected to create strong outliers, making this an ideal system in which to identify genes that have evolved under selection. Selection can be identified and invoked for outlier loci, even if these genes only show relatively small frequency differences. The strong finding of this work is that genes with muscle-related functions are over-represented among all the 87 loci identified as outliers (20 muscle-related genes, 23%). To us, this is an elegant way of demonstrating that differentiation is not solely a consequence of plasticity, and points the way forward towards an estimate of how much of the phenotypic difference is genetic, something that may become more widely used in studies of geographical variation. In this study, Qu *et al.* actually held sparrows in a common garden environment in the lowlands for a month, noting that differences in muscle mass were maintained, but adding multiple caveats about the short duration of the experiment and sample sizes.

Why has muscle changed and is it hypoxia or the cold temperature that has caused this (or something else)? Long ago, C.H. Waddington stated: ‘The species has to use whatever genes are at its disposal to meet the demands of selection’ [4]. When placed in a new environment, standing variation is immediately available for selection, expected to shift allele frequencies at all segregating loci that contribute to traits affecting fitness. It is perhaps a little easier to see why standing variation associated with climate is present in lowland individuals, given trade-offs between flight performance and heat conservation (muscle shivering is an important means of generating heat in birds) and the geographical variation in climate that the species has experienced for much of its evolutionary history. Qu *et al.* think otherwise and suggest that flight-muscle differences in the Tibetan plateau are associated with high-altitude adaptation. However, they do note that the larger fiber area observed in the flight muscle of highland birds increases the distance oxygen has to travel through the cell, which seems inconsistent with this idea. Their study shows how we might contrast alternatives, using multiple directions of research, from studies of fitness variation in the wild to mechanistic evaluation of both the genes and the phenotypes, and assessment of parallel evolution across other species and locations.

***Conflict of interest statement***. None declared.
